# Concerning Medical Students

**Published:** 1886-11-27

**Authors:** 


### CONCERNING MEDICAL STUDENTS.

THE clinical lectures at Aberdeen Royal Infirmary began a fortnight ago. Dr. Will, on Friday, 29th Oct., led off with some interesting cases of his own; he spoke emphatically of the need among students of attending to the common cases of practice, to the important little requisites of every-day manipulation, and, in a word, to the niceties of minor surgery as against the well-known management of major operations. Dr. Garden followed on Tuesday, 2nd November, and in a few pointed introductory remarks dealt sharply with the fault of our ordinary medical teaching—namely, the tendency to general instruction by book and lecture, without the proper corrective of clinical work. We get plenty of science, but little practical wisdom. He endorsed the words of Mr. Timothy Holmes—our teaching is too "demonstrational." His aim would be to bring the students actually face to face with all the varieties of disease in his power. Dr. Ogston, on Friday, 5th Nov., had a series of bed-side examinations and note-takings, followed up by short demonstrations of the cases. The experiment was very successful. Undoubtedly clinical work is the most varied, educational, and charming part of a medical student's career. For the speculative man, no less than for the practical, there is no end of variety and interest, besides those much treasured chances of the willing lover of human nature, so dear to the true student, for

"His little, nameless, unremembered acts
Of kindness and of love."

